# Supplementary figures and images for: Ethnically biased microsatellites contribute to differential gene expression and glutathione metabolism in Africans and Europeans
Source: PLoS One. 2021 Mar 25;16(3):e0249148. doi: 10.1371/journal.pone.0249148 (PMC7993785; doi:10.1371/journal.pone.0249148)

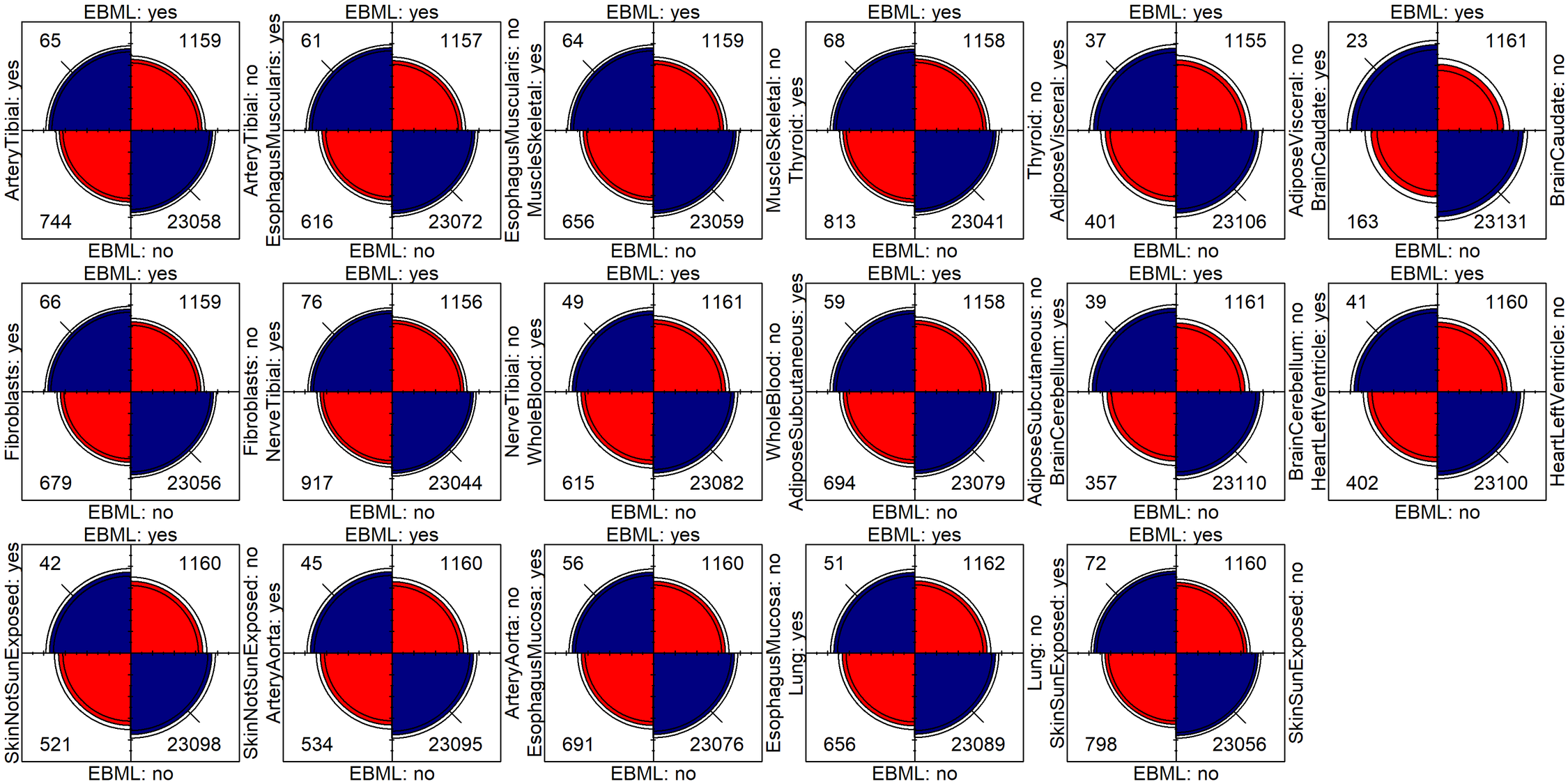

Supplement: S1 Fig — (TIF) [file pone.0249148.s001.tif]

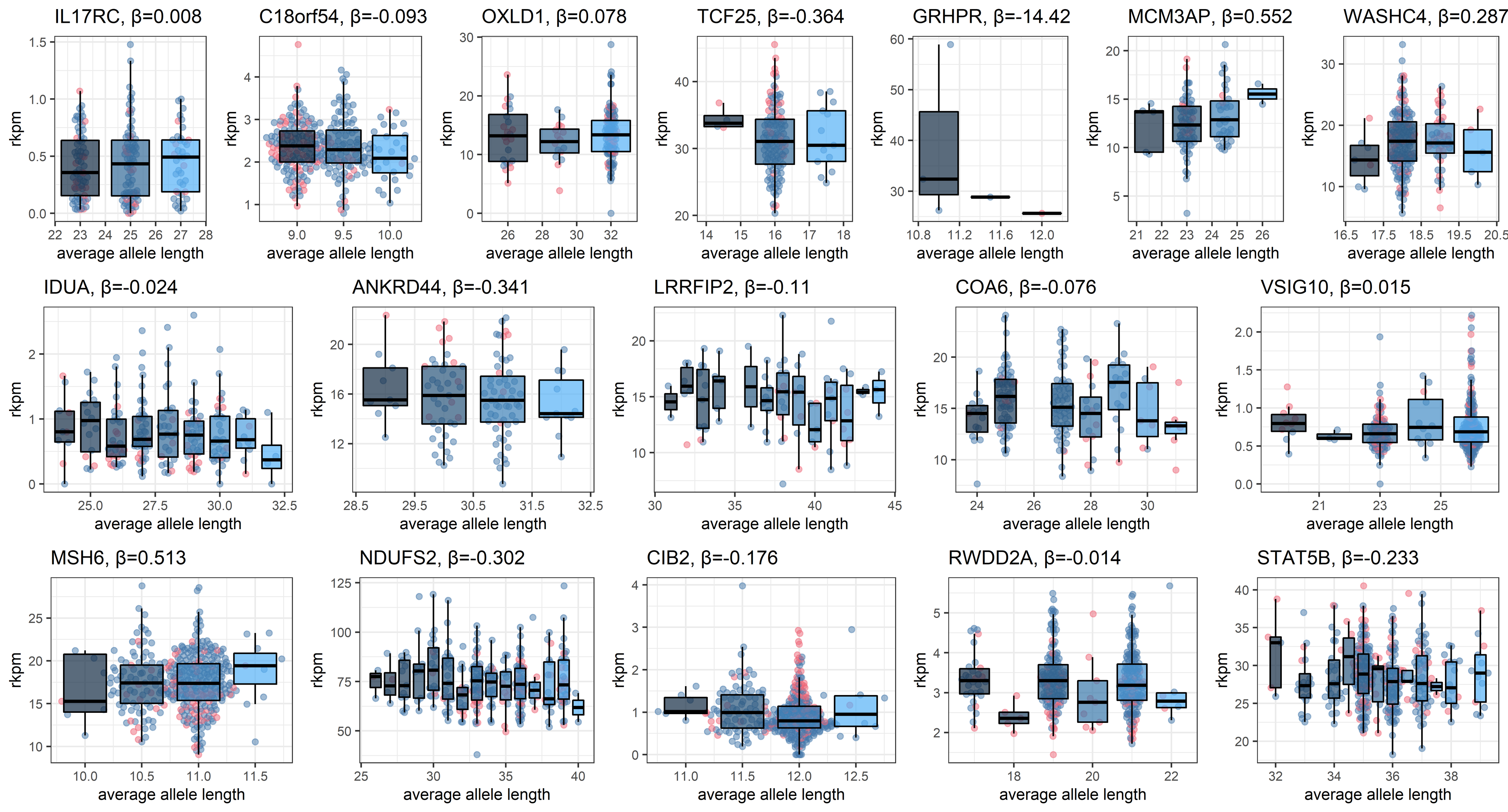

Supplement: S2 Fig — (TIF) [file pone.0249148.s002.tif]

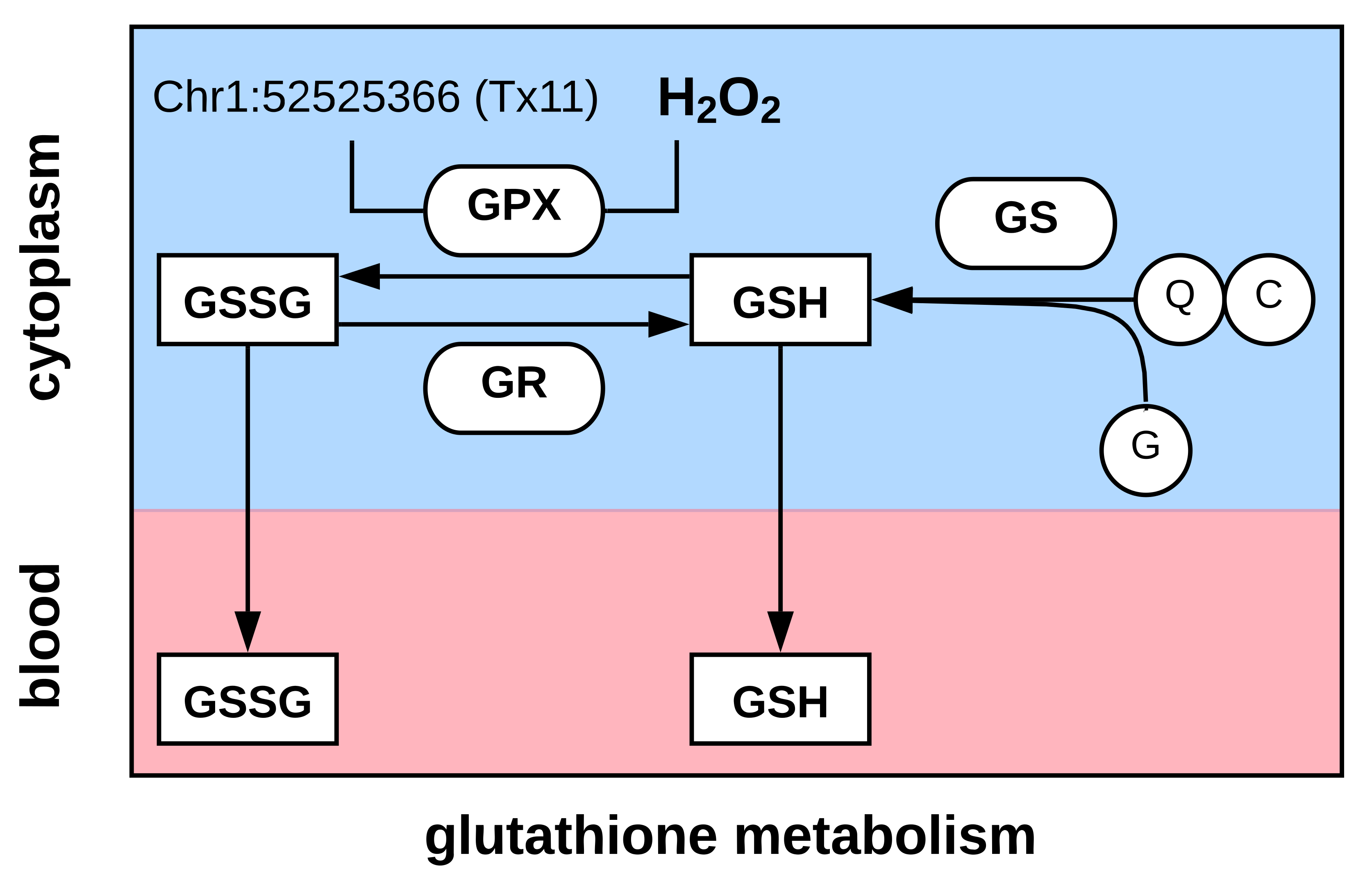

Supplement: S1 Code — (ZIP) [file pone.0249148.s008.zip › DockerizedEBML/data/fig5.png]

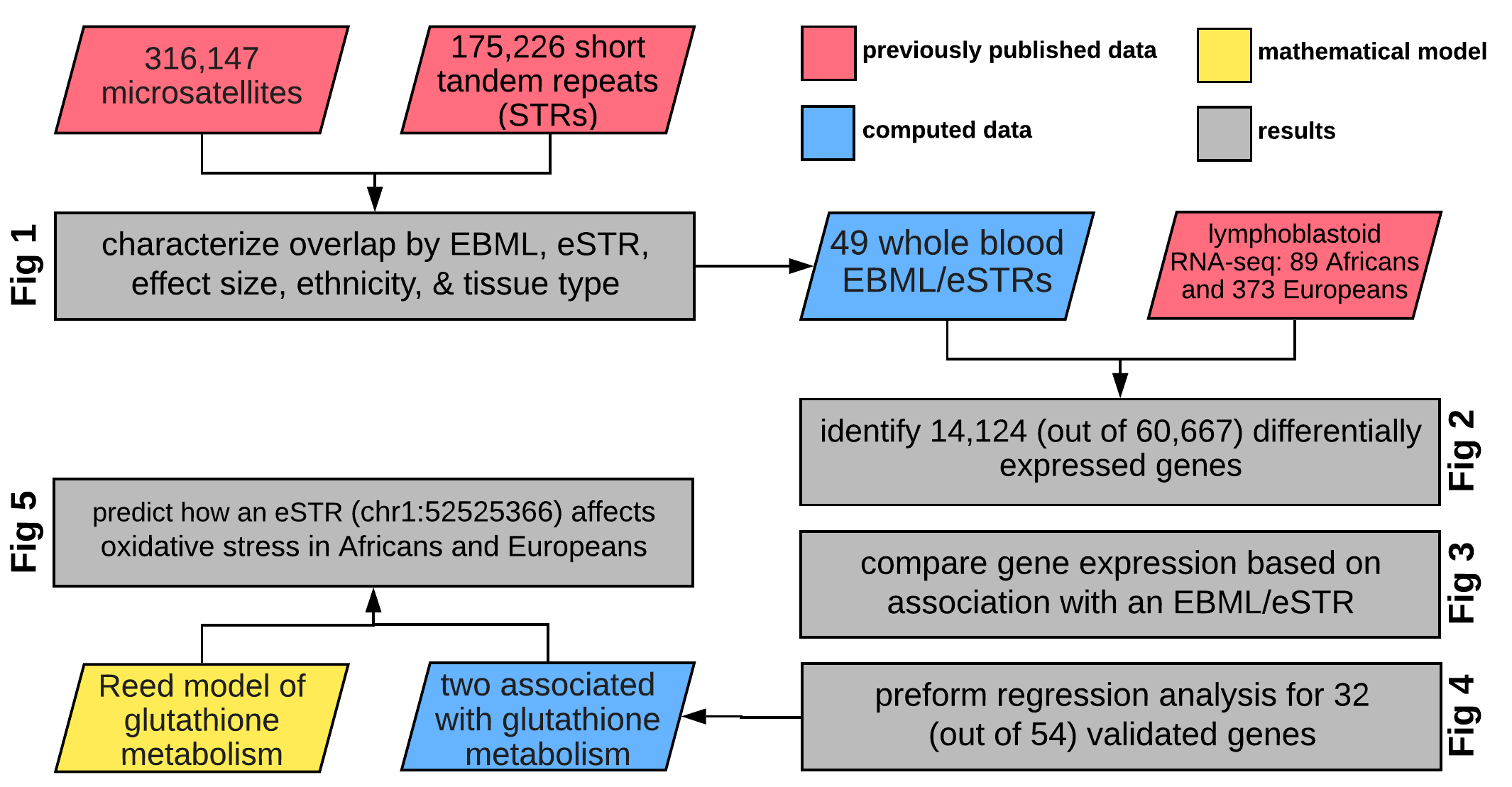

Supplement: S1 Code — (ZIP) [file pone.0249148.s008.zip › DockerizedEBML/data/fig6.png]
