# Supplementary material for: Ethnically biased microsatellites contribute to differential gene expression and glutathione metabolism in Africans and Europeans
Source: PLoS One. 2021 Mar 25;16(3):e0249148. doi: 10.1371/journal.pone.0249148 (PMC7993785; doi:10.1371/journal.pone.0249148)
Supplement: Attachment — Submitted filename: Response to Reviewers.pdf [file pone.0249148.s009.pdf]

Edward Via College of Osteopathic Medicine
VIRGINIA • CAROLINAS
AUBURN

Primary Care Research Network
Center for Bioinformatics and Genetics
Nick Kinney, PhD
2265 Kraft Drive
Blacksburg, Virginia 24060
Phone: 301.338.1181
Email: nkinney06@gmail.com

February 26, 2021

Dr. Danillo G Augusto
Academic Editor of PLOS ONE
RE: POINT BY POINT RESPONSE TO PEER REVIEW COMMENTS (1ST REVISION)

Dear Dr. Augusto:

In the pages that follow we respond point-by-point to each of the reviewer comments concerning our manuscript: "**ETHNICALLY BIASED MICROSATELLITES CONTRIBUTE TO DIFFERENTIAL GENE EXPRESSION AND GLUTATHIONE METABOLISM IN AFRICANS AND EUROPEANS**". The reviewer concerns were relatively minor but nonetheless have improved our manuscript. Reviewer one suggested elaborating on the caveats of the datasets used in our analysis. In response, we have added a new discussion paragraph and revised several passages throughout the text. The changes have improved the work and we thank both reviewers for their feedback. We would also like to clarify sources of funding as requested by the journal:

- The authors received no specific funding for this work.
- The funders had no role in study design, data collection and analysis, decision to publish, or preparation of the manuscript.
- The authors did not receive a salary from any funders.
- The authors received no specific funding for this work.

We are excited to share our revised manuscript with the scientific community and look forward to hearing back. There are no related articles published or under consideration. If there are any specific questions that need a conversation, my cell is 301 338-1181.

Sincerely,

Nicholas Kinney

Dr. Nicholas Kinney
Primary Care Research Network and the Center for Bioinformatics and Genetics, VCOM
Professor of Biomedicine, VCOM

Editor:

Please clarify the similarities and differences of this current submission compared to the previously published manuscript by the same authors: 10.1371/journal.pone.0225216. Please highlight the differences in the aims and also the novelty this current submission brings compared to the previous manuscript. If necessary, please amend the text to clarify this point.

Our previous publication (10.1371/journal.pone.0225216) in this field had four main results. First, we identified 3,984 ethnically biased microsatellite loci (EBML). Second, we showed that a statistically significant number of EBML coincide with known eSTRs; i.e. they affect gene expression. Third, we demonstrated association between EBML, core matrisome genes, and matrisome associated genes. Fourth, we found a significant number of EBML in putative selective sweep regions. Overall, these findings were observational in nature.

This submission pursues questions related to how EBML affect gene expression, i.e. questions that are functional in nature. How many EBML affect gene expression? What is the impact of EBML on gene expression relative to other differentially expressed genes? Can we infer how EBML affect phenotype using mathematical models? The limitations of our previous study and novelty of the current study are summarized in paragraphs 7 and 8 of the introduction:

> Our [previous] findings showed that EBML and eSTRs possess mutual overlap suggesting that microsatellites contribute to differential gene expression in human populations (21). However, this possibility was not rigorously validated with gene expression data. Furthermore, the association was established prior to the discovery of 28,375 eSTRs in 2019 (10). We hypothesize that a combination of EBML, newly discovered eSTRs, and gene expression data (RNA-seq) can be used to show that microsatellites contribute to differential gene expression and phenotype in human populations.

The novelty of the current work is also highlighted in the discussion. Briefly, we demonstrate how RNA-seq can be used to validate and quantify the effects of EBML/eSTRs on gene expression. In addition, we demonstrate that mathematical models can be repurposed to infer how EBML/eSTRs affect phenotype. The latter point has broader implications. In particular, there are over 28,000 known eSTRs and nearly 1,000 biological systems models for human available in databases such as JWS online and EBI BioModels. Thus, we anticipate phenotypic effect of many more eSTRs can be made using our approach on existing mathematical models.

We agree with the editor that it is important to distinguish how the two manuscripts differ. Although the differences can be found in the introduction and discussion, we have appended a short paragraph to the methods section making these differences explicitly clear.

Reviewer 1:

I would like to invite the authors to reflect and discuss the impact of using these datasets on the results obtained:

The reviewer makes important points regarding the challenge of integrating the three main datasets we consider: (a) EBML identified from KGP samples, (b) eSTRs identified from GTEX samples, and (c) RNA-seq data from GEUVADIS consortium. The common thread of our response is that there is a “discovery bias” which favors identification and subsequent validation of EBML/eSTRs in Europeans. Critically, this does not invalidate any of our results or affect reproducibility. In what follows we elaborate on each dataset and our effort to integrate these datasets.

(i) EBML are identified from 5 main ethnic groups (data set 1KG - AFR, EUR, SOA, EAS, AME);

First, we consider the “discovery bias” for EBML. Our previous publication identified 3,171 EBML for African populations and 450 EBML for European populations. However, these EBML were discovered using relatively small samples of sequenced individuals: 667 Africans and 502 Europeans. Given these samples sizes, we can say with confidence that the true number of EBML exceeds 450 for Europeans and 3,171 for Africans. Despite the greater number of African EBML, the percent of true EBML discovered is probably greater for Europeans. A passage from our previous paper justifies this speculation:

> the 1000 Genomes Project falls short in capturing genetic diversity in some populations. Principal component analysis has shown that genetic diversity in Southeast Asia is under-represented in the 1000 Genomes Project [1]; and, whole genome sequencing of African Populations has revealed millions of unshared genetic variants [2].

Essentially, the 1000 Genomes Project captures more of the genetic diversity in Europeans than other ethnicities: particularly Africans and Asians. Estimating the true number of EBML for Europeans and Africans would probably require sophisticated power analysis beyond the scope of this work. In any case, the true number of EBML is not necessary for the current work. It is more important to recognize that the 450 EBML for Europeans and 3,171 EBML for Africans are all valid despite a larger number of undiscovered African EBML. Our results now emphasize that there are likely a disproportionate number of undiscovered EBML for African and Asian populations (see the second section of our results).

1. Lu D, Xu S. Principal component analysis reveals the 1000 Genomes Project does not sufficiently cover the human genetic diversity in Asia. Front Genet. 2013;4:127. Epub 2013/07/13. pmid:23847652; PubMed Central PMCID: PMC3701331.

2. Sherman RM, Forman J, Antonescu V, Puiu D, Daya M, Rafaels N, et al. Assembly of a pan-genome from deep sequencing of 910 humans of African descent (vol 51, pg 30, 2018). Nat Genet. 2019;51(2):364–. WOS:000457314300025.

(ii) eSTR are identified from the GTEX database, which presents an ethnic bias, where the majority of the samples that they integrate are declared “white” (~ 85%);

Superficially, the predominately white/European samples in the GTEX database seem to favor the discovery of European eSTRs; however, this incorrect as there is no notion of “European” eSTRs or “African” eSTRs. Any repeat shown to affect gene expression – an eSTR by definition – in Europeans likely also affects gene expression in Africans. Of course, there are exceptions; specifically, neutral repeats in Africans could hypothetically be eSTRs in Europeans and vice versa. These hypothetical repeats could be explained by standard concepts in evolution such as migration, genetic drift, and selection. Consequently, it is possible that some of the 28,000 eSTRs discovered from the GTEX database are neutral (false positives) in Africans; and some of the GTEX neutral repeats could be eSTRs (false negatives) in Africans. The number of such exceptions is difficult to estimate.

Probably, most of the 28,000 known eSTRs would be reproduced in an exclusively African cohort. Thus, despite the GTEX samples being predominantly white, we do not expect the set of 28,000 eSTRs to have a significant bias towards Europeans or Africans.

(iii) Data from RNAseq (GEUVADIS consortium) are only available for a 1KG subsample of Africans and Europeans.

We use RNA-seq data (GEUVADIS consortium) to compare whole exome gene expression in cohorts of 89 Africans and 373 Europeans. First, we identified 14,124 (out of 60,667) differentially expressed genes. These numbers would certainly change with a more balanced cohort; however, the differentially expressed genes we report are not necessarily bias towards Europeans or Africans. Expression for all of them clearly differ in the cohorts we compare. The differentially expressed genes included 32 (out of 54) associated with a whole blood EBML/eSTRs. Statistically speaking, this supports our hypothesis that some EBML contribute to differential gene expression.

As the reviewer points out, it is important to consider how the aforementioned “discovery bias” for EBML may influence this result. To reiterate, there are undoubtedly undiscovered EBML for EUR populations and even more for AFR populations. We can have confidence in our result – that EBML/eSTRs are associated with differential expression – by preforming the following hypothetical exercise.

Start with the original contingency table used to test association between EBML/eSTRs and differentially expressed genes (note the numbers sum to 60,667). A chi-squared test confirms EBML are associated with differential expression (p=1.08e-9; χ^2 test). This calculation is found in the caption to figure 2 of our manuscript.

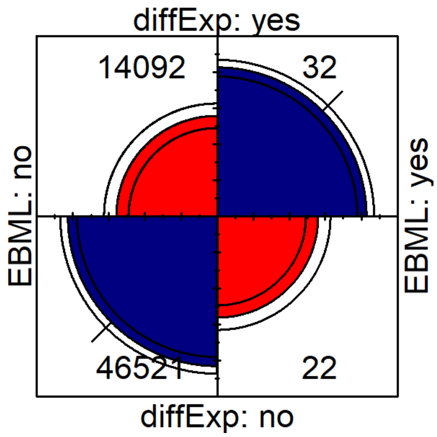

Next, suppose we discover 14 new EBML/eSTRs for Europeans and 94 new EBML/eSTRs for Africans. In other words, whole blood EBML have tripled from 54 to 162. Let’s further assume that only 25 of the new EBML are associated with a differentially expressed gene, i.e. they are drawn randomly from the left column of 14,092 differentially expressed genes and 46,521 similarly expressed genes above. We tally a new (hypothetical) contingency table shown below. A chi-squared test would still indicate that EBML are associated with differential expression (p=4.7e-4; χ^2 test).

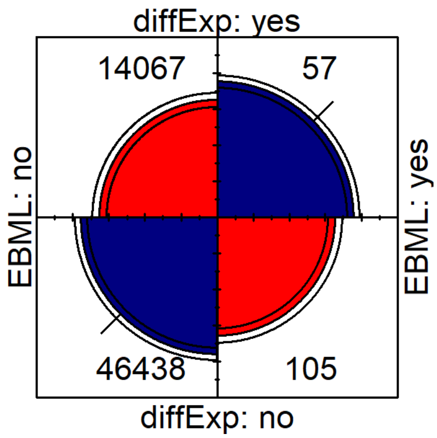

What if we hypothetically discover 216 new whole blood EBML increasing the original 54 by a factor of 5? Again, we draw the 216 randomly from the left column of 14,092 differentially expressed genes and 46,521 similarly expressed genes. A chi-squared test would still indicate that EBML are associated with differential expression (p=.007; χ^2 test).

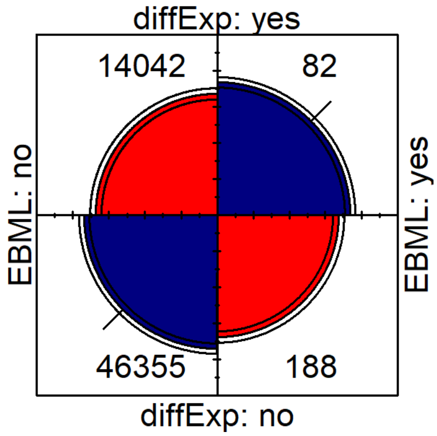

Overall, the association we report between EBML/eSTRs and differentially expressed genes seems robust to the possibility of numerous undiscovered EBML and eSTRs (even if these undiscovered EBML are random with respect to gene expression). It is unfortunate that suitable RNAseq data was only available for two ethnicities (AFR and EUR); but such data would probably reiterate once again an association between EBML/eSTRs and differentially expressed genes.

Why did the authors not previously filter the EBML for AFR and EUR, since confirmatory analyses can be performed only for these groups?

This is an astute detail! We thank the reviewer for careful reading of our manuscript. Indeed, we pursue confirmatory analysis for 49 EBML that were not explicitly filtered with respect to AFR and EUR populations. If we had filtered prior to confirmatory analysis we would have found 7 EBML for EUR and 37 EBML for AFR with three overlapping (EBML in both populations). In other words, filtering would have eliminated 8 of 49 EBML we considered. We provide links to the genetic tables for these 8 EBML:

http://www.cagmdb.org/view_table.php?id=162059
http://www.cagmdb.org/view_table.php?id=387118
http://www.cagmdb.org/view_table.php?id=305630
http://www.cagmdb.org/view_table.php?id=347723
http://www.cagmdb.org/view_table.php?id=492319
http://www.cagmdb.org/view_table.php?id=496611
http://www.cagmdb.org/view_table.php?id=548113
http://www.cagmdb.org/view_table.php?id=584568

Let’s take a look at genotypes for the EBML at chr16:89850472 (first link above):

chr16:89850472-89850487

genotype	AFR	EUR
13|16	11	4
16|16	382	156
16|19	1	16
19|19	1	0

p-value 1.39e-7

It may come as a surprise that genotypes for this short tandem repeat (STR) differ in Africans and Europeans. However, this difference alone is not sufficient to call this region an EBML in either population. For each EBML, at least one ethnicity must have genotype frequencies statistically different from the remaining four. Examining the complete tally of genotypes for this region reveals mutual similarities between AFR and SAS as well as AMR and EUR (table below). In fact, this region is only a rigorous EBML for EAS populations. But, given the difference between African genotypes and European genotypes, we feel it is appropriate to keep this region for confirmatory analyses.

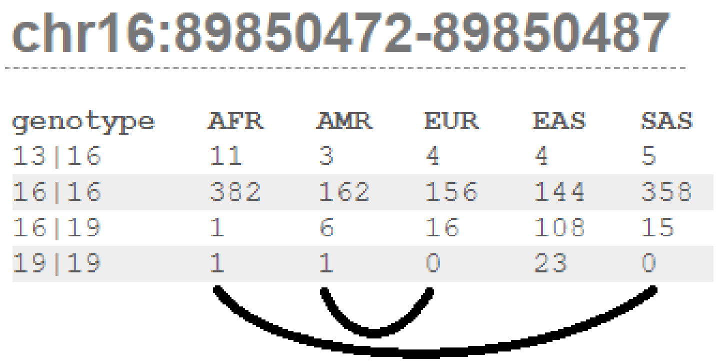

chr16:89850472-89850487

genotype	AFR	AMR	EUR	EAS	SAS
13|16	11	3	4	4	5
16|16	382	162	156	144	358
16|19	1	6	16	108	15
19|19	1	1	0	23	0

We could make a similar case for keeping more of the eight regions in our confirmatory analysis; we opted to retain all eight for simplicity. Indeed, all eight regions show some differences in AFR and EUR populations; and it is very unlikely that these eight fringe cases affected our analysis. For example, our previous response demonstrates robustness of association between EBML/eSTRs and differentially expressed genes.

How can the ethnic bias of GTEX samples favor the identification of EBML/eSTR EUR?

The reviewer makes an important point that the GTEX samples – used to discover eSTRs – are predominately European. For the most part we will reiterate our response from above. Essentially, repeats shown to affect gene expression – eSTRs by definition – in Europeans should also affect gene expression in Africans. Of course, there will be rare exceptions; but we expect the majority of the 28,000 known eSTRs to be reproducible in an exclusively African cohort.

The identification of EBML has a more obvious “discovery bias” stemming from the KGP samples. In particular, the KGP samples under-represent African genetic variation. Consequently, relatively fewer EBML have been established for Africans (3,171 total) than Europeans (450 total). But it is important to emphasize that most of our analysis focuses on a set of 49 EBML with clear genetic differences between the two populations (7 EBML for EUR, 37 EBML for AFR, and 8 polymorphic regions discussed above). Even if most of the undiscovered EBML are unique to AFR populations – as we suspect – our analysis of these specific 49 does not change.

Perhaps the one piece of analysis that is affected by the KGP and GTEX datasets is the tally of 313 regions across ethnicity and tissue type. Taking into consideration the reviewer’s question, figure 1D is misleading because it directly compares raw numbers of EBML by ethnicity. With more balanced samples – particularly KGP samples – the African, South Asian, and East Asian bars would represent more EBML regions and be even larger. In fact, under-representation of Africans and South Asians is well established for the KGP [1,2]. The figure is correct, but only for discovered EBML. We have added a short passage to this section of the manuscript that clarifies the issue.

1. Lu D, Xu S. Principal component analysis reveals the 1000 Genomes Project does not sufficiently cover the human genetic diversity in Asia. Front Genet. 2013;4:127. Epub 2013/07/13. pmid:23847652; PubMed Central PMCID: PMC3701331.

2. Sherman RM, Forman J, Antonescu V, Puiu D, Daya M, Rafaels N, et al. Assembly of a pan-genome from deep sequencing of 910 humans of African descent (vol 51, pg 30, 2018). Nat Genet. 2019;51(2):364–. WOS:000457314300025.

How many of the 313 are exclusively AFR and EUR? (maybe this info is in table S3, but it has not been made available for review)

Yes, the intersection of ethnicity, tissue type, EBML, and eSTRs is summarized in table S3. Although this information is not directly available for review, we have already released a dockerized jupyter notebook to reproduce all figures and allow others to further investigate our results:

https://github.com/nkinney06/dockerizedEBML

Tabulating the 313 regions by ethnicity reveals 242 (AFR) and 51 (EUR) EBML, respectively. The manuscript subsequently focuses on 49 Whole Blood EBML/eSTRs. These include 7 EBML for EUR and 37 EBML for AFR with three overlapping (EBML in both populations).

A section with caveats would be welcome for the study.

We have added a paragraph to the discussion recognizing several caveats pointed out by the reviewer. We elaborate on some of these below.

(i) Discussion of challenges to obtain reliable allele calls for microsatellites from NGS low coverage data

Indeed, microsatellite allele calls remain challenging; particularly from NGS low coverage data. Mapping issues are common for large indels and realignment around indels is typically required before genotype calling. Our previous work on EBML (10.1371/journal.pone.0225216) used two strategies to increase sensitivity of allele calls; specifically, requirements were placed on (a) sequencing depth and (b) sequence logos.

(a) Requirements placed on sequencing depth. Allele calls were only made for regions with six or more mapped reads; consequently, most of the alleles in our call sets are supported by three or more reads. This strategy was implemented to mitigate the effects of PCR artifacts and read duplication.

(b) Requirements placed on sequence logos. We defined sequence logos for each microsatellite by concatenation of its 3' flanking sequence, repeat unit, and 5' flanking sequence. We used 5bp for the flanking regions. For example, microsatellites 'GCTGC(A)^{34}CTTAG' and 'GCTGC(A)^{15}CTTAG' received the same sequence logo: 'GCTGCACTTAG'. Only microsatellites with unique sequence logos were considered in our analysis. This strategy was implemented to mitigate occurrences of microsatellite polymorphisms mapping incorrectly to regions with similar (or identical) sequence logos.

We are open to including a further discussion of challenges; however, no allele calling was actually preformed in this work. We use the allele calls from our previous work: 10.1371/journal.pone.0225216. Thus, it could be confusing if we delve into the challenges of allele calling in this work. As a compromise, we mention that there are challenges and encourage the reader to pursue details in our previous work that made the calls [1].

1. Kinney, N., Kang, L., Eckstrand, L., Pulenthiran, A., Samuel, P., Anandakrishnan, R., Varghese, R.T., Michalak, P. and Garner, H.R., 2019. Abundance of ethnically biased microsatellites in human gene regions. *PloS one*, *14*(12), p.e0225216.

(ii) Limitations for RNAseq comparison and quantification of gene expression (mapped reads x NGS low coverage)

We agree it is a good idea to remind the reader that RNA-seq has limitations. Measurement noise inevitably arises due to random sampling inherent in the assay [1]. This complicates differential expression analysis, particularly for between sample comparison of genes with low transcription rate. Measurement noise is exacerbated by heterogeneity of sequencing platforms and analysis protocols [1]. Thus, sequencing coverage, platform, sample sizes, and analysis could all increase the rate of false positives.

The RNA-seq analyses preformed in our work mitigates – but does not eliminate – these issues. First, the data is relatively homogeneous. Publication of the GEUVADIS samples is self-described as "the first uniformly processed high-throughput RNA-sequencing data from multiple human populations with high-quality genome sequences" [2]. Second, we use the industry standard analysis pipeline – DESeq2 – to identify differentially expressed genes from count data [3]. Our code is available at https://github.com/nkinney06/dockerizedEBML. Third, regression analysis (figure 4 of our manuscript) is preformed on RPKM normalized counts. The differentially expressed genes we identify undoubtedly still contain false positives; however, association between EBML/eSTRs with those differentially expressed genes appears to be very robust (see our response above).

1. SEQC Consortium, 2014. A comprehensive assessment of RNA-seq accuracy, reproducibility and information content by the Sequencing Quality Control Consortium. *Nature biotechnology*, *32*(9), p.903.

2. Lappalainen, T., Sammeth, M., Friedländer, M.R., Ac't Hoen, P., Monlong, J., Rivas, M.A., Gonzalez-Porta, M., Kurbatova, N., Griebel, T., Ferreira, P.G. and Barann, M., 2013. Transcriptome and genome sequencing uncovers functional variation in humans. *Nature*, *501*(7468), pp.506-511.

3. Love, M., Anders, S. and Huber, W., 2014. Differential analysis of count data–the DESeq2 package. *Genome Biol*, *15*(550), pp.10-1186.

(iii) As shown in other studies, many EMBL and eSTR are found in regions with genetic signatures of natural selection. In this sense, it is important to discuss the possible hitchhiker effect on microsatellites (causal variant x tag variant).

Our previous publication on EBML (10.1371/journal.pone.0225216) did recognize this possibility. A passage from the results of that publication states the following:

> "it is possible that EBML are simply in linkage disequilibrium with targets of selection such as nearby SNPs. Overall, it seems likely that some EBML–particularly the ones that overlap eSTRs–have bona fide adaptive potential."

In fact, the 49 EBML/eSTRs we investigate here have already been scrutinized to infer possible hitcher effects [1]. Essentially, causality of eSTRs was estimated using CAVIAR [2]: a type of statistical fine-mapping. On average, 12.2% of eSTRs had higher causality scores than all nearby SNPs. At the same time STR-SNP linkage disequilibrium is approximately half of the traditional SNP-SNP LD [3]. Needless to say, hitchhiker effects remain challenging to sort out. Perhaps it is most accurate to say that the 49 EBML/eSTRs contribute to gene expression along with nearby SNPs. We have added a short passage to our results (second section) that recognizes these caveats.

1. Fotsing, S.F., Margoliash, J., Wang, C., Saini, S., Yanicky, R., Shleizer-Burko, S., Goren, A. and Gymrek, M., 2019. The impact of short tandem repeat variation on gene expression. *Nature genetics*, *51*(11), pp.1652-1659.

2. Hormozdiari, F., Kostem, E., Kang, E.Y., Pasaniuc, B. and Eskin, E., 2014. Identifying causal variants at loci with multiple signals of association. *Genetics*, *198*(2), pp.497-508.

3. Payseur, B.A., Place, M. and Weber, J.L., 2008. Linkage disequilibrium between STRPs and SNPs across the human genome. *The American Journal of Human Genetics*, *82*(5), pp.1039-1050

At various points throughout the text, especially in the results section, the authors state that the partially presented results support the hypothesis that microsatellites contribute to the expression's differential. This is repetitive and is not necessary.

Agreed. It is difficult to strike the ideal balance between clarity and being too repetitive. We have made the manuscript more economical in a few places.

Title: the authors have confirmed data and analyses for differential expression and GPX7 only for African and European populations. For this reason, I suggest that the title of the study is not so comprehensive and is restricted to these two population groups;

Also agreed. We have updated to a more appropriate title:

> ETHNICALLY BIASED MICROSATELLITES CONTRIBUTE TO DIFFERENTIAL GENE EXPRESSION AND GLUTATHIONE METABOLISM IN AFRICANS AND EUROPEANS

line 147 .. "Tabulating the 313 candidate regions…"

In retrospect this passage seems redundant. It has been re-worded:

> The 313 candidate regions include abundant…

According to figure 1d, nerve tibial genes have greater overlap for the total of the 5 super-populations. If we look only at Africans it seems to me that the thyroid is bigger. Please consider revising the sentence.

This passage was potential confusing. It now reads as follows:

> The 313 candidate regions include abundant overlapping EBML/eSTRs associated with nerve tibial and thyroid genes (Fig 1d). Tabulating by ethnicity reveals abundant EBML in Africans…

Reviewer 2:

This is a very well-conducted work. Data sounds relevant and worthy.

We thank the reviewer for the positive feedback.
